# *Biology direct:* celebrating 7 years of open, published peer review

**DOI:** 10.1186/1745-6150-8-11

**Published:** 2013-04-30

**Authors:** Eugene V Koonin, Laura F Landweber, David J Lipman

**Affiliations:** 1 National Center for Biotechnology Information, National Library of Medicine, National Institutes of Health, Bethesda, MD 20894, USA; 2 Department of Ecology and Evolutionary Biology, Princeton University, Princeton, NJ, 08544, USA

## 

*Biology Direct*, an online open access journal published by BioMed Central, is celebrating its 7th anniversary. *Biology Direct* started as an experiment, perhaps a daring one, on a new system of open peer review, under which the signed reviews and the author responses are published as an integral part of the final version of each article. The goals of the journal were set high: we strived to establish a new system of peer review that we hoped would avoid the all too obvious pitfalls of anonymous peer review. In addition, we expected that *Biology Direct* would generate productive scientific debate that would substantially add to the content of an article, in particular by alerting readers to potential problems with the reviewed work as well as additional relevant data and ideas [1,2].

Now, 7 years after the journal’s launch, we can conclude that the new approach to scientific publication works, despite difficulties and failures along the way. Here are some telling numbers. Over 7 years, the journal published 365 papers that have been accessed over a total of 2,000,000 times (as of April 2013). In 2012 *Biology Direct*’s Impact Factor exceeded 4, which puts it ahead of numerous well-established, highly respected professional journals. Two articles have been cited more than 100 times each, making them citation classics, and 13 articles have been cited more than 50 times each.

More important than these numbers is the informal feedback from colleagues. Numerous discussions with scientists in diverse fields, primarily in various branches of quantitative biology, support the impression that *Biology Direct* has become a well-known forum, above all for constructive, open discussion of new results and ideas. Often, the exchange with reviewers emerges as the most interesting part of a *Biology Direct* paper, and the section to which some readers turn first. This was one of the goals of *Biology Direct* from its inception, and, some growing pains notwithstanding, we believe this goal has been reached.

In addition, articles in *Biology Direct* have been recognized twice in BioMed Central’s Annual Research Awards, firstly with Nick Lane’s article on the origin of eukaryotes in 2011 [3], and recently with Geoffrey Diemer and Kenneth Stedman’s 2012 paper describing their discovery of a novel virus genome from an extreme environment [4].

*Biology Direct* finished its 7th year on a strong note, but the Editors think that in order to progress further, changes are due. It was never our intention to make *Biology Direct* a large scale publishing operation, similar to, for instance, *PLOS One*. The philosophy of *Biology Direct* is not to publish every scientifically sound paper, as *PLOS One* does, but rather to publish a relatively small collection of papers, some of which report major discoveries but all are sufficiently interesting and perhaps even provocative to stir productive discussion. That said, we would like to substantially increase the number of papers published and openly reviewed in this way, and to do so without compromising the quality of the articles, but rather via diversification of the subjects that are covered in the journal and we hope enhancing the impact of the articles at the same time.

To this end, *Biology Direct* will be undergoing a re-organization. The journal started by publishing papers in the fields of computational biology, genomics and systems biology; the mathematical biology and immunology sections were later added. To date, the journal clearly owes its success to the “core” areas it covers (namely, evolutionary genomics, systems biology and bioinformatics). It appears plausible that, at this time, the model of published, open peer review appeals more strongly to and is more suitable for authors of primarily non-experimental research articles and for research in new areas that combine massive data collection with extensive computational analysis, such as systems biology or metagenomics.

Taking into account the experience of the first 7 years, we have decided to expand the scope of *Biology Direct* around its core areas, hence the reorganization of the journal into 7 sections:

– bioinformatics

– genomics

– evolutionary biology

– structural and molecular biology

– non-coding DNA and RNA

– systems biology

– mathematical biology

The new sections, each run by two editors, will replace the current Genomics, Bioinformatics and Systems Biology sections and incorporate the current Mathematical Biology section. Each of these sections, in addition to full length Research Papers, Review, Hypotheses and Opinions, will also publish brief Discovery Notes, thereby subsuming the current Discovery Notes section. The Immunology section is being discontinued, with some of its members joining other sections. To a large extent, the Editorial Board membership for the new sections of *Biology Direct* is already in place, and a strong group of new Editors is currently recruiting an expanded cadre of Editorial Board members.

We are confident that, in the rapidly expanding universe of scientific publication, *Biology Direct* will continue to fill its unique niche as a forum for open peer review and discussion for years to come, and the journal will very likely introduce new sections as we go. It is also our goal that the journal further raises its profile, publishing some of the most innovative, original papers in quantitative biology. Above all, this optimism derives from the strength of the group of the Section Editors and Editorial Board members at *Biology Direct* that, in our view, reflects an strong vote of confidence from the scientific community.
